# Supplementary material for: Dynamic Vascular Imaging Using Active Breast Thermography
Source: Sensors (Basel). 2023 Mar 10;23(6):3012. doi: 10.3390/s23063012 (PMC10057499; doi:10.3390/s23063012)

Supplementary Materials

Patient 282: Left-sick. Location: inner lower quadrant.

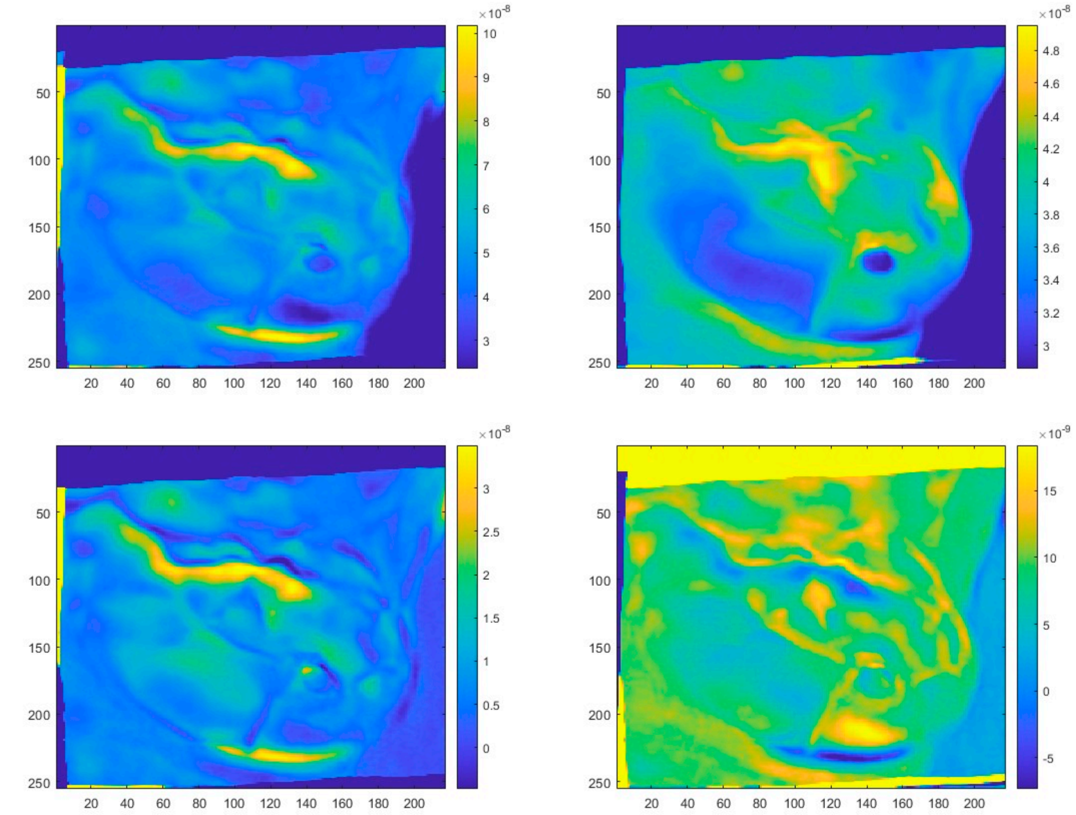

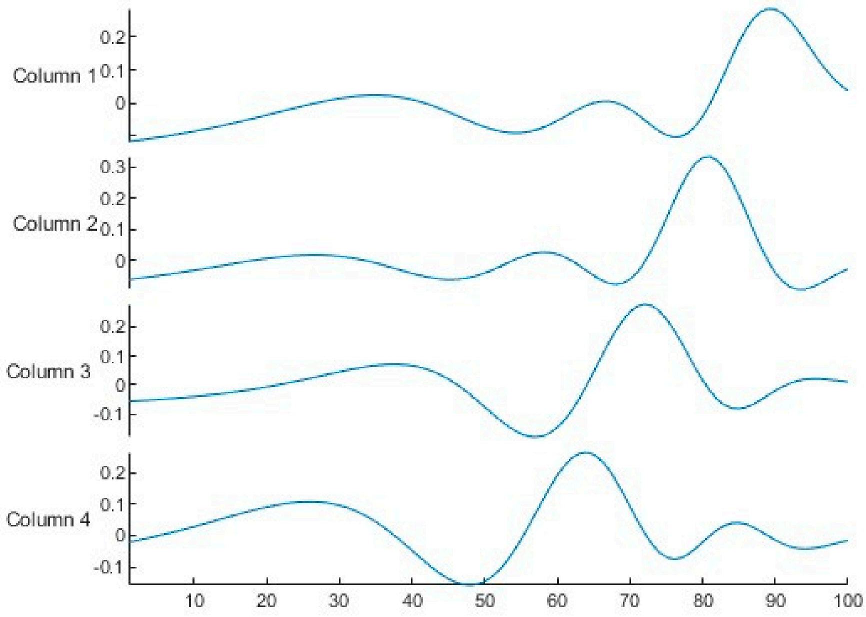

Supplementary material Patient 282: Right-healthy.

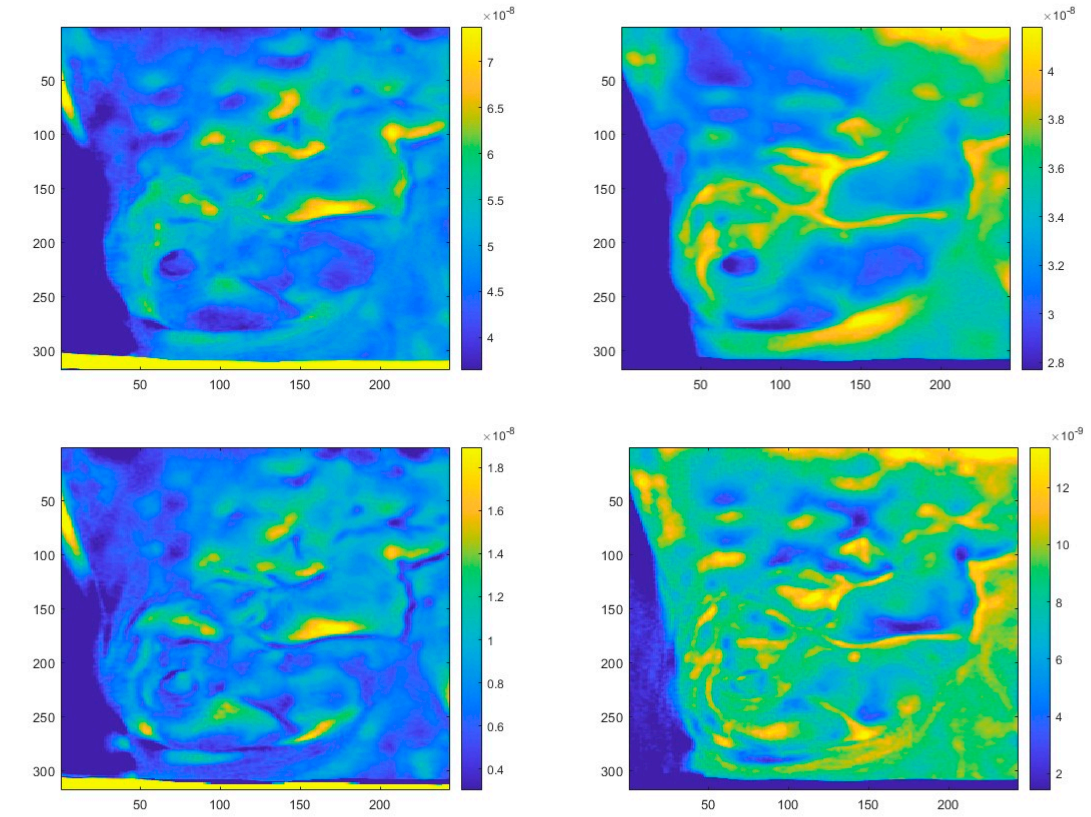

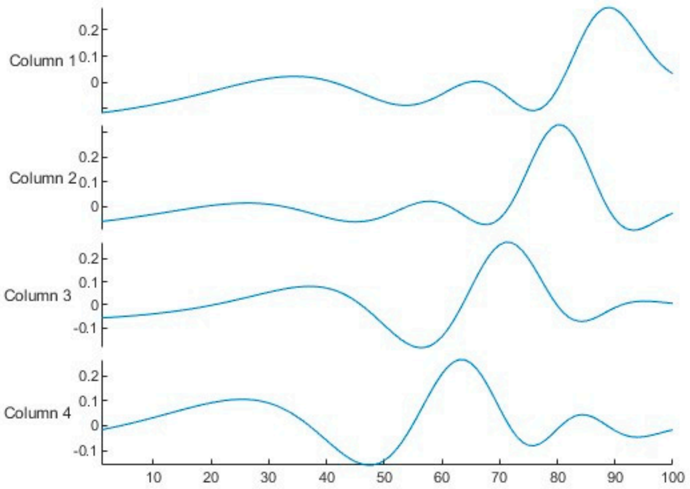

Supplementary material Patient 285: Left-healthy.

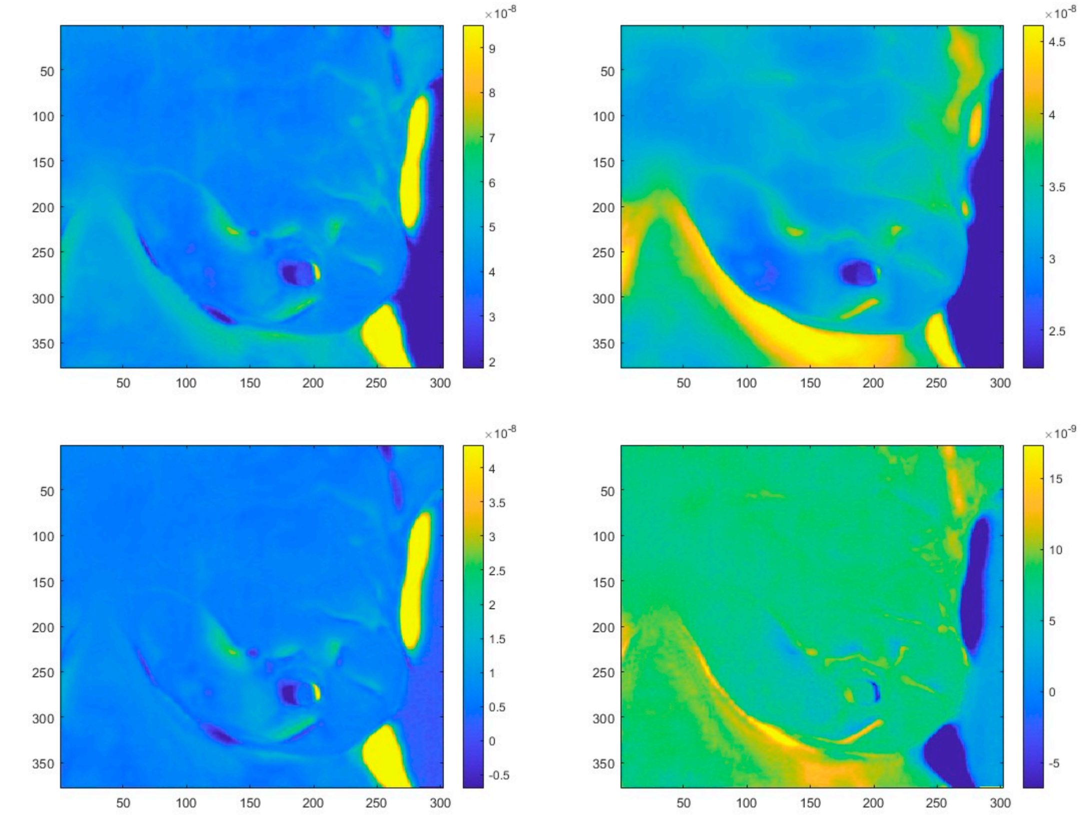

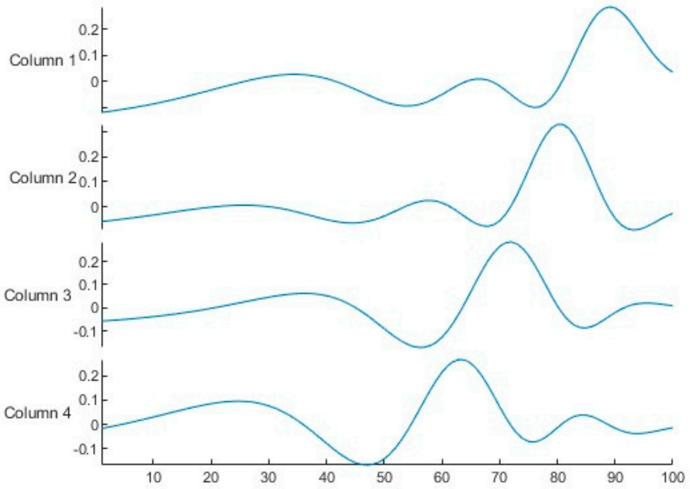

Patient 285: Right-sick. Location: outer lower quadrant.

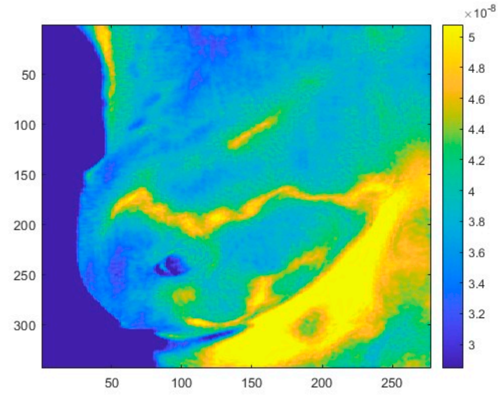

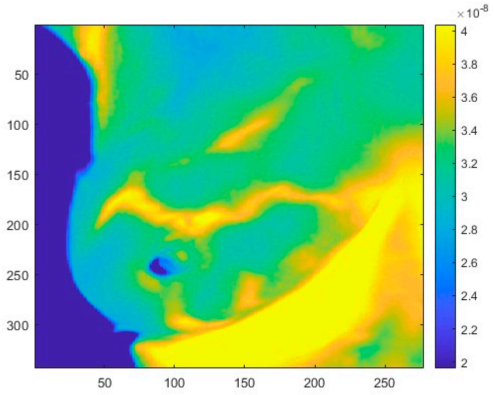

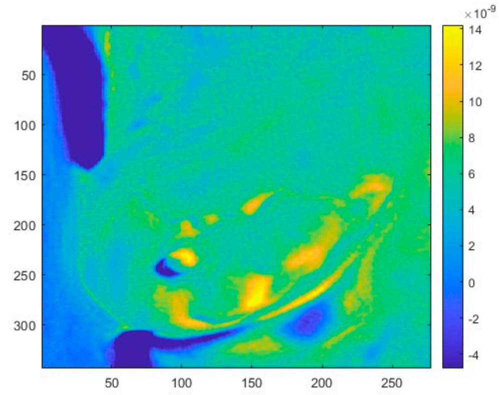

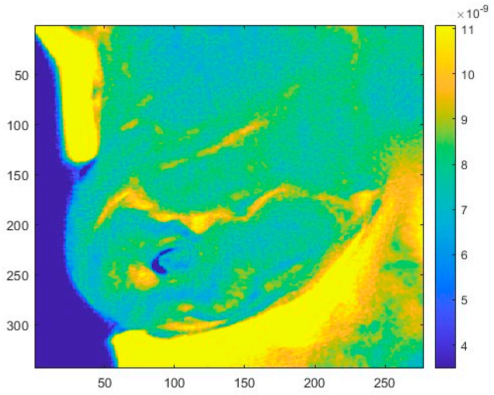

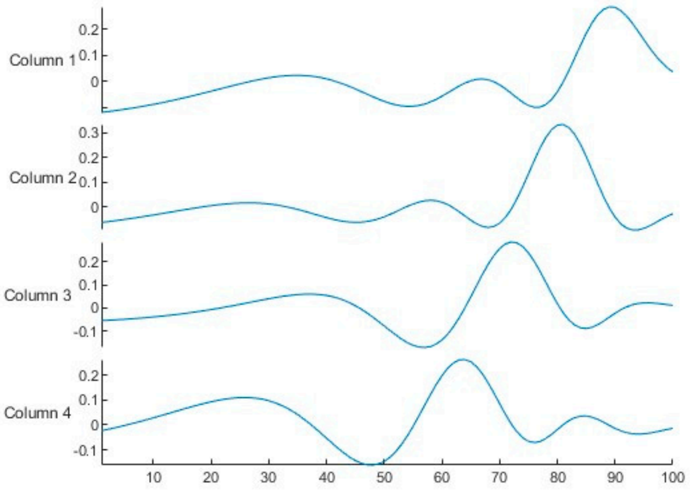

Supplementary material Patient 286: Left-sick. Location: upper outer quadrant.

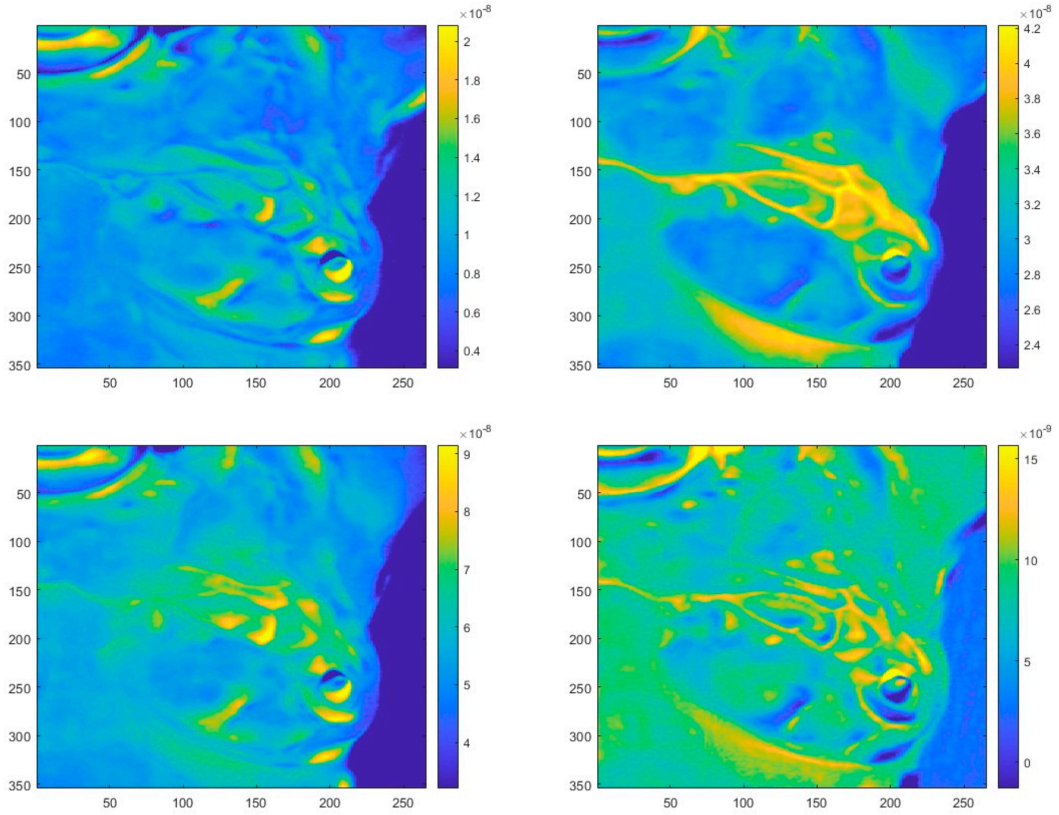

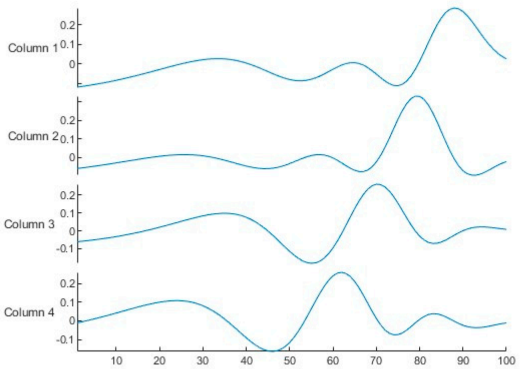

Patient 286: Right-healthy.

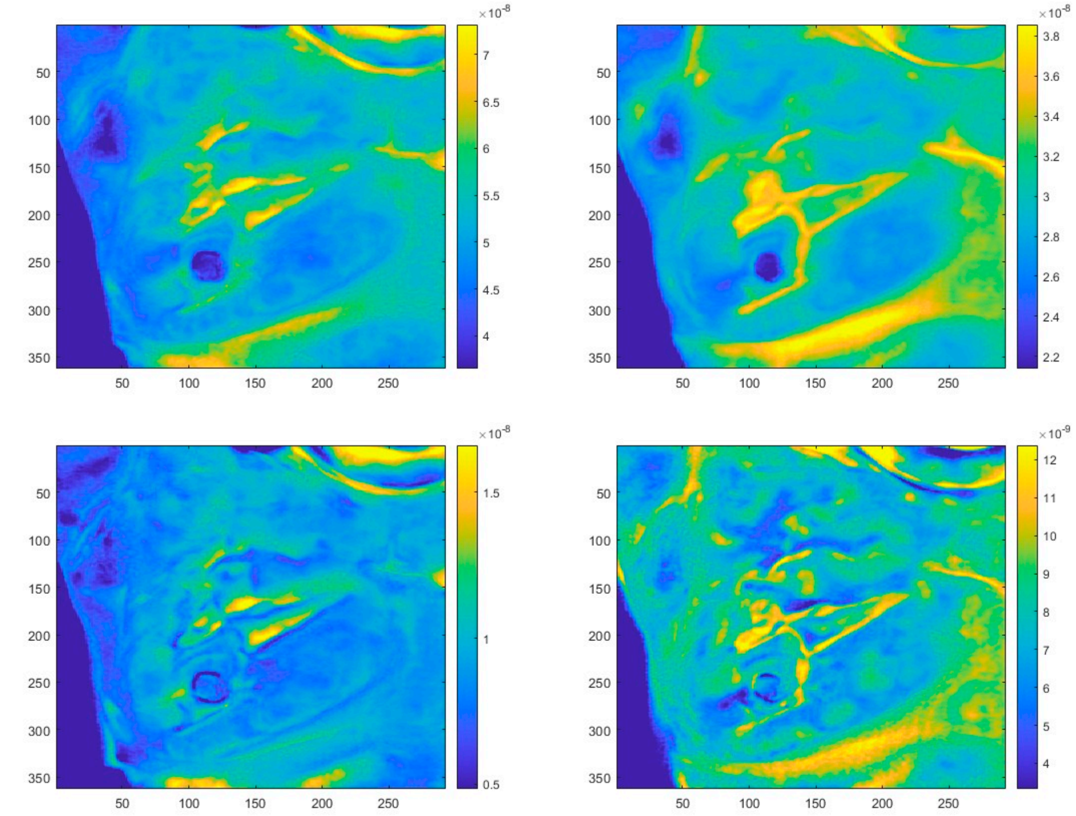

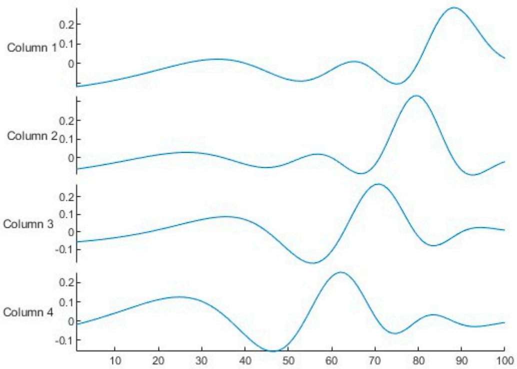

Supplement: Supplementary file 1 [file sensors-23-03012-s001.zip › sensors-2247556-supplementary.pdf]
